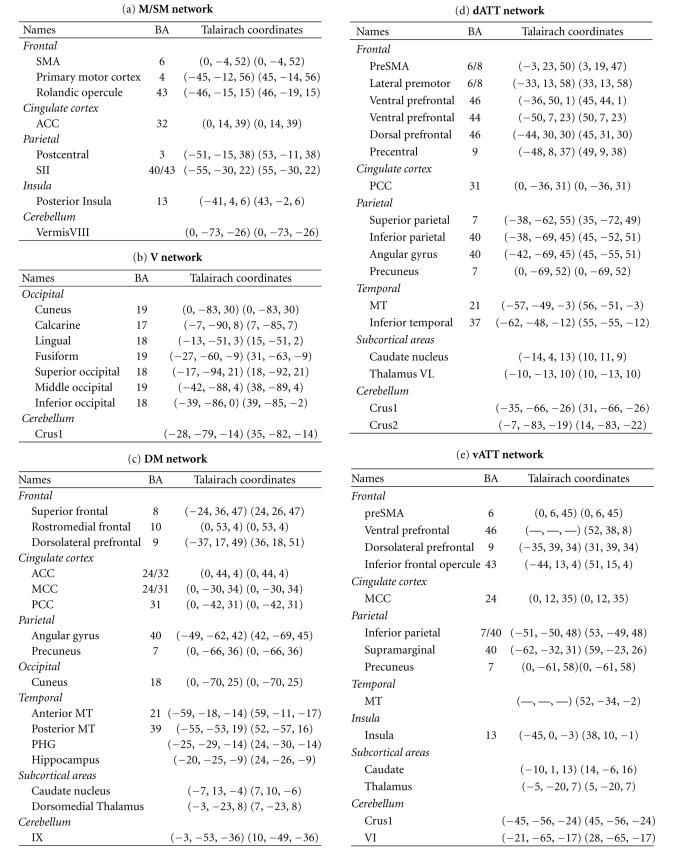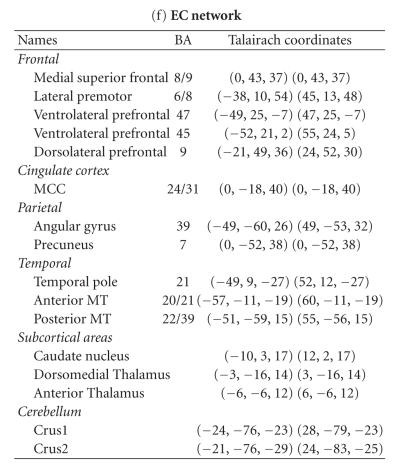# Contribution of Exploratory Methods to the Investigation of Extended Large-Scale Brain Networks in Functional MRI: Methodologies, Results, and Challenges

**DOI:** 10.1155/2008/218519

**Published:** 2008-05-08

**Authors:** V. Perlbarg, G. Marrelec

**Affiliations:** ^1^U678, Inserm, Paris 75013, France; ^2^Faculté de Médecine Pitié-Salpêtrière, Université Pierre et Marie Curie, Paris 75013, France

## Abstract

A large-scale brain network can be defined as a set of segregated and integrated regions, that is, distant regions that share strong anatomical connections and functional interactions. Data-driven investigation of such networks has recently received a great deal of attention in blood-oxygen-level-dependent (BOLD) functional magnetic resonance imaging (fMRI). We here review the rationale for such an investigation, the methods used, the results obtained, and also discuss some issues that have to be faced for an efficient exploration.

## 1. INTRODUCTION

Blood-oxygen-level-dependent (BOLD) functional magnetic resonance imaging (fMRI) is an imaging technique that makes it possible to dynamically and noninvasively track metabolic and hemodynamic changes in the brain [1, 2]. The early developments of BOLD fMRI data analysis have mostly relied on a method called general linear model (GLM), whose objective is to pinpoint the differential involvement of certain regions during various tasks [3–5]. Voxel clusters that exhibited such a behavior are declared “activated” and gathered into a so-called activation map that provides the output of the GLM approach; each map represents all regions that are significantly correlated with the stimulus time course. GLM-based methods have been extensively used in order to extract regions in a wide variety of conditions (see, e.g., [6] for a review of activation studies related to the premotor cortex).

The GLM, however, does not properly render the brain's intricate organization, which is believed to be based on two major principles: segregation and integration [7, 8]. According to these two principles, functional tasks are performed by specific collections of brain regions, also called networks, that are anatomically connected and can engage in complex interactions [9–11]. Even though the BOLD contrast is only remotely related to neuronal activity, it was first hypothesized, and then evidenced, that this imaging modality is able to reflect, at least to some extent, the strong constraints imposed on the brain by segregation and integration. This realization came from the investigation of the (misleadingly called) “rest” condition. First, studies showed that brain regions could still be correlated at rest, hinting for the existence of functional brain networks that could still be present and imaged even when no task was explicitly required from the subject [12, 13]. Network investigation also started with a closer examination of the “baseline,” that is, the signal measured when a subject is in the “rest” condition of a protocol, between two task conditions [14]. This approach was justified from the fact that, from an energetic perspective, the brain uses a significant part of the body's energy, independently of the presence or absence of a “task” [15, 16]. As methods of increasing complexity were developed and validated, the objective of many methodological developments shifted from GLM-related procedures to methods that were able to extract networks from BOLD fMRI data.

This paper is an attempt to review the latest advances in investigation of extended large-scale networks in fMRI from a methodological perspective, as well as the networks that have been found using these methods. This new methodology, if confirmed, has very deep implications in terms of methods that should be developed, and we discuss some of the issues that such methods will have to deal with in order to provide reliable and useful results.

## 2. LARGE-SCALE NETWORKS AS A CONSEQUENCE OF BRAIN ORGANIZATION

An extended large-scale functional brain network may be defined as a (potentially large) number of segregated regions (potentially spread over the whole brain) that interact in order to execute a coherent task. While Bressler and Tognoli [17] mostly consider “high-level brain functions of which cognition is comprised,” it is important to emphasize the fact that about any function that the brain is able to process is likely to have a network representation (see, e.g., Section 4 for a description of some low-level brain functions, such as vision and audition processing).

Large-scale networks share many important features. First, they are widely distributed over the brain. As a consequence of the segregation principle, it is hypothesized that they can be broken down into small brain regions, coined “nodes” by Mesulam [18], “units” by Marrelec et al. [19], and “local cortical area networks” by Bressler and Tognoli [17], each region being characterized by a consistent functional behavior. Such nodes can readily be identified in subcortical structures, which are often gathered into nuclei [20]. As to the cortex, despite cytoarchitectonic features (embodied, e.g., by the work of Brodmann [21] and his eponymous regions) that vary across its surface, its parcellation based on structural criteria alone remains globally a challenge. Nonetheless, local brain areas are also strongly characterized by their function [17]. For instance, primary sensory regions (e.g., visual) have been localized in a quite reproducible manner; within these regions, areas responding more specifically to certain types of inputs have been successfully identified (e.g., vertical versus horizontal lines in the primary visual cortex [22]). Still, even though various levels of specialization can usually be observed, there is a general agreement that most regions cannot be unambiguously associated with one specific function (see, e.g., [23] for Broca's area) and, in general, a region will exhibit a certain level of “multifunctionality” [17]: its contribution will not be limited to one task but will be allowed to vary within a given range of functions that it is able to implement.

As a consequence of the integration principle, large-scale networks are also characterized by potentially distant regions with strong (anatomical) connections and (functional) interactions. Whether top-down or bottom-up, serial or parallel, connections and interactions are quintessential of networks [18, 24, 25]. Anatomically, interregional connectivity is suspected to be rather sparse [26–30]. Even though most connections originating from one region are thought to re-enter the same region, axons are known to connect regions that are far apart from each other, for example, homologue regions [e.g., [31–33]]. Functionally, these connections have translations at all levels, from electrophysiology [34–38] to measures of the electromagnetic field [39] and of the BOLD signal [12, 13].

Whether coined “new phrenology” [40] or considered as being “beyond phrenology” [25], such an approach leads to a model of brain functions in which most functional tasks are subserved by functional brain networks, that is, collections of specialized regions that collaborate in order to generate a coherent behavior [11, 36]. In support of this approach, several networks have already been described and documented. Luria [10] refers to three blocks: one that “regulates the energy level and tone of the cortex,” another one that is strongly implicated in information processing, and a last one that is involved in higher, complex tasks, such as “the formation of intentions and programs for behavior.” Mesulam [18] proposed two distinct subdivisions of the brain. First, based on the co-occurrence of functions with similar features, the brain can be divided into five major “subtypes”: primary sensory-motor, unimodal association, heteromodal association, paralimbic, and limbic. There are also at least five large-scale networks, each dealing with a specific cognitive function: spatial awareness, language, explicit memory/emotion, face-object recognition, and working memory-executive function. These networks are not isolated from one another, but interact in very complex fashion, for example, through “transmodal” areas.

## 3. fMRI INVESTIGATION OF LARGE-SCALE NETWORKS

Relying on the assumption that BOLD fMRI is indeed able to image brain networks (see, e.g., [15] for a review of the neurophysiological substrate of neuroimaging), two categories of methods may be identified for such studies: approaches that make use of prior cognitive information and fully exploratory methods.

### 3.1. Using neurocognitive information

Correlational methods were historically the first ones to be applied to investigate large-scale networks in fMRI data analysis, in the form of functional connectivity studies and functional connectivity maps [12, 41–47]. Starting from a voxel or region—the so-called “seed” voxel/region—one extracts all voxels whose time courses are significantly correlated with that of the seed. Measures other than temporal correlation have also been used, such as coherence and partial coherence [48, 49]. Selection of the seed region is a key issue in studies of functional connectivity. First, a brain region is selected according to its function (e.g., cortical representation for hand movement, [47]). The corresponding seed is then obtained from either prior anatomical knowledge or functional manipulation. Anatomically, common approaches consist of using coordinates in a standardized space (Talairach or MNI) [44], or having an expert delineate the region on anatomical images [48]. Functionally, the seed can be obtained from an activation map, provided that the region of interest can be characterized by its implication in a specific task (e.g., the primary motor cortex in a simple movement) [41, 45, 47].

As opposed to effective connectivity—where Gonçlaves and Hall [50] showed that results of SEM analyses may vary depending on the choice of the seed voxel—robustness of functional connectivity maps with regard to the selection of the seed region and its spatial extent have barely been examined yet. Vincent et al. [51] showed that, for the visual or the somatomotor network, the resulting functional connectivity map was robust to the choice of the starting seed region. Many other parameters (e.g., design, size of each region) may have an influence on the outcome of the analysis, potentially leading to different spatial structures or correlation values between structures. Full exploration of a whole network (i.e., with many regions) would imply the recourse to several successive computations of functional connectivity maps, each map being used to select a region significantly correlated as seed voxel for the next step—a procedure that is lengthy, complex, and whose convergence is not assured. Wang and Xia [52] have recently proposed a method to perform this exploration in only one step.

### 3.2. Blind exploration

The goal of fully exploratory methods is to provide data-driven approaches of large-scale network detection in which no prior cognitive information is required for the methods to proceed. A number of such procedures have been proposed, most of them relying more or less closely on either of the two key features of large-scale networks, namely integration and segregation.

The vast majority of approaches proceed as follows. Based on a similarity measure, they gather voxels irrespective of their anatomical proximity (and, hence, of segregation) into separate classes that are strongly similar to each other and dissimilar from one another. For each class, the output is a map representative of the class and an associated time course. All methods have one or several parameters whose tuning affects the number of classes. Since each class tends to gather voxels that are strongly correlated, it is often univocally identified with a large-scale network. Blind approaches include methods based on eigenvalue decomposition, such as principal component analysis (PCA) [53–55], correlation clustering [56], Kendall's coefficient of concordance [57, 58], K-means [59, 60], fuzzy clustering methods [54, 56], self-organizing map algorithms [61], Kohonen clustering neural network and fuzzy C-means [62], hierarchical clustering [60, 63], integration and information-theoretic quantities [64, 65], and spatial independent component analysis (sICA) [66]. While most methods provide maps that are exclusive (a voxel can only belong to one map), a few (e.g., fuzzy clustering or ICA) provide an index of the plausibility for a voxel to belong to each of the different classes. Most methods also provide local criteria, calling for stepwise analyses, at the exception of PCA and ICA that use global measures and, consequently, are able to perform classification in one step.

Most approaches mentioned in the previous paragraph have only been used a limited number of time in fMRI data analysis so far. This can probably be accounted for by the complexity of their algorithms, which is commensurate with the difficulty of the task at hand. Outstandingly, sICA has been used quite a lot recently, with results that are rather promising. Regardless of its popularity, though, the network interpretation of the results obtained needs to be proved beyond simple criteria (these include, e.g., that voxels located close to each other or in homologue regions tend to belong to the same class). For instance, for PCA, Friston and Büchel [67] mention that the interpretation of the eigenimages in biological terms might be dubious, since they could be rotated in the data space and still be a solution to the problem (but see [68]). By contrast, components obtained through ICA can be more easily related to known physiological noises or functional processes [66, 69]. The methodological reasons for this success are, however, still not clear, and many explanations are plausible: the relevance of the assumption of spatial independence, the adequacy of the underlying mixing model, the efficiency of the global criterion/one-step discrimination approach, or some interesting feature of the information-theoretic optimization algorithm. In any case, the fact that its application simplifies the results to a maximum and produces a very limited number of widespread networks, making interpretation easier, clearly plays in its favor (compare, e.g., with [70, 71], or [72]). Its sensitivity, which is much higher than that of clustering methods, might also explain its success. Nevertheless, it must still be kept in mind that the assumptions underlying ICA (perfect synchrony within a network and spatial independence between networks) impose an extreme and unrealistic case of integration. While the simplification of several time courses into one is performed only once for ICA, the stepwise procedures implemented by other methods essentially go through the same approximation at each step, leading to an error that is probably far larger.

Unlike the numerous approaches to functional integration, few methods have specifically sought to extract segregated regions. Some methods decrease the complexity of the data by using predefined regions (e.g., according to the Tzourio-Mazoyer et al. [81] template). Approaches using predefinite regions do not check that all voxels within a region exhibit homogeneous bahaviors; they merely assume that it is the case. Average signals are then extracted from each region, on which any integration-based approach, such as hierarchical clustering [71, 72], can be applied. Intuitively, many clustering methods mentioned previously (e.g., K-means, hierarchical clustering, or information-theoretic measures) could easily be applied for the purpose of detecting segregated regions by incorporating a constraint of contiguity between voxels that could be merged. Among these, only the information-theoretic approach explicitly takes both within- and between-classes measures of similarity into account. Specifically, they optimize a so-called functional clustering index (FCI) that keeps a balance between region homogeneity (strong segregation) and sparseness of inter-regional interactions (low integration) [64, 65]—the latter constraint being hard to justify from a network perspective. As to other clustering methods, as noted by Goutte et al. [60] in accordance to Huygens' formula, maximizing a measure of the internal coherence of a class (associated with segregation) is often equivalent to minimizing the same measure of coherence but computed between classes (that could be associated with integration). As such, the behavior of such methods with regard to networks would again lead to questioning.

A tentative approach to consider simultaneously segregation and integration has been conducted by the large scale network identification (LSNI) method by Bellec et al. [80]. LSNI first clusters neighboring voxels into small regions using a region-growing algorithm [82] and then selects regions that exhibit a significant correlation with other distant regions. Such a procedure allows to define brain functional regions and networks in a purely data-driven way. While the sensitivity of the algorithm proposed was rather low, it had the great advantage to explicitly define and address the two principles of functional segregation and integration. So far, this method seems adapted for individual analyses; its extension to group studies seems limited due to the subject-dependent definition of regions.

## 4. TYPOLOGY OF NETWORKS EXTRACTED WITH fMRI

During the last decade, several brain systems have been studied in fMRI using functional connectivity-related approaches. These studies have revealed integrated systems, including primary systems and associative networks. Exploratory approaches have also allowed to extract several functional networks at once. Even if all brain areas are not included in a network, these networks involve many areas and constitute a possible functional parcellation of the brain.

The motor network was the first network studied through functional connectivity analyses. Biswal et al. [12] reported correlations in low-frequency resting-state fluctuations between left and right motor areas using single-slice fast-sampled acquisitions. This result was later reproduced with multislice acquisitions where an extended motor network was shown to correlate with a region in the primary motor cortex [44, 47]. Lowe et al. [44] showed that other functional networks could be detected using other seed regions, namely the visual network with a seed around the calcarine fissure and a limbic network with a seed in the amygdala. An auditory and a language systems were later extracted in the same way [41, 43]. Other networks were also studied using the seed-region functional connectivity approach, such as the default-mode network [83–86], the attentional networks [42, 83, 87], and memory networks [46, 88].

**Figure 1:**
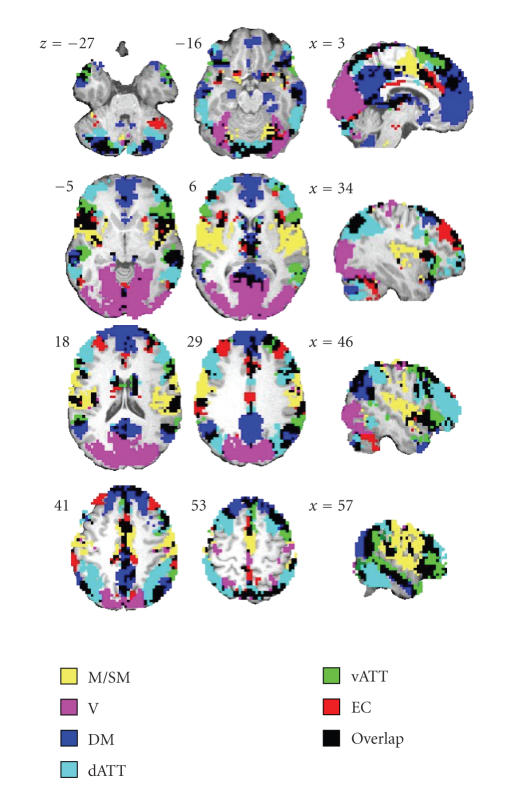
Example of extended large-scale networks extracted in fMRI at rest. The six networks were identified using spatial ICA and a hierarchical clustering approach similar to that of [79] on a group of 20 healthy subjects acquired at rest. Networks—M/SM: motor/sensorimotor, V: visual, DM: default mode, dAtt: dorsal attentional, vAtt: ventral attentional, EC: executive control. The auditory network was not found as a separate network but some temporal regions of the primary auditory cortex are overlapped by other networks (in particular the M/SM network and the two attentional networks). The union of all networks does not add up to comprise the entire brain; for instance, some parts of the frontal cortex do not belong to any reported networks. By contrast, some brain regions simultaneously belong to several networks (in black).

More recently, a larger number of functional networks were revealed using exploratory methods based on ICA [76–78]. Even if the number of extracted networks varied, their spatial organizations were reproducible across studies. For instance, all three studies just mentioned found functional networks involving the same systems that were sometimes split into different parts (e.g., left/right, rostral/caudal). Using group ICA studies of resting-state datasets [76–78], which were the most reproducible, we selected seven functional networks: a motor/sensorimotor system, a visual system, an auditory system, a default-mode network, a dorsal attentional network, a ventral attentional network, and an executive control network. Van de ven et al. [75] systematically studied the reproducibility of ICA results on individual datasets and the results using hierarchical clustering [63] and self-organizing map algorithm (SOM) [61] were presented on an individual level. The results from studies that provided a systematic description of all networks found are reported in Table 1. In Figure 1 and Table 2, we also reported results from a study on a population of 20 healthy subjects acquired at rest, where networks were extracted using spatial ICA and a hierarchical clustering approach similar to that of Esposito et al. [79].

The sensorimotor system involves at least the pre- and postcentral gyri (including the primary motor cortex and supplementary motor area). The visual system involves both medial striate and extrastriate regions (calcarine sulcus and lingual gyrus), as well as lateral occipital regions (nonprimary visual regions); these two visual networks (primary and associative) were identified separately by the three group-ICA studies. The auditory system involves principally lateral superior temporal gyri, the Heschl's gyrus and insular cortex. The so-called default-mode network involves the anterior and posterior cingulate cortices, the medial prefrontal cortex and lateral parietal regions [15, 89–91]. The dorsal attentional network involves lateral prefrontal and dorsal partietal cortex; these regions are involved in visio-spatial control [42, 92, 93]. The ventral attentional network involves inferior occipito-parietal regions and inferior lateral prefrontal regions; these regions are principally involved in new item recognition [42, 94]. Last, the executive control network involves superior and middle prefrontal cortices, ventrolateral prefrontal cortex and anterior cingulate gyrus [95].

Other studies have applied these exploratory approaches to extract integrated functional networks from fMRI datasets acquired during external stimulation [73, 79, 80]. The results of these three studies are also compiled in Table 1. These results showed that the detection of the functional networks not directly related to the stimulation were less sensitive than that without external stimulation.

## 5. FUTURE ISSUES

Tracking the presence of extended large-scale networks in BOLD fMRI data raises many issues. We have here focused on some aspects related to the neurocognitive aspects of networks, their identifiability by fMRI, and the methodological questions raised by network analyses.

### 5.1. Neurocognitive aspects

The major issue to be faced is arguably the very definition of an extended large-scale brain network. Indeed, even though the brain is far from being fully connected, any region is eventually connected to any other regions if one takes polysynaptic connections into account. Obviously, stating that the brain can be considered as one network is by no way satisfying, no more than it is to say that each macrocolumn forms a networks by itself. The strongly hierarchical nature of the brain's anatomical and structural organization induces similar characteristics at all levels. As the brain can be decomposed in networks, each network can in turn be further partitioned into subnetworks, subnetworks into subsubnetworks, and so on. Furthermore, even though there probably is a (potentially loose) relationship between anatomical and functional organizations, it is still unknown how functional integration and segregation are coded in anatomical terms. For the exact same structural organization, it has been shown that networks can be observed to break down as one discriminates different sets of functional tasks or behaviors with increasing precision. For instance, the visual system can be decomposed into a ventral and a dorsal stream [22]. These two subnetworks, albeit interacting, have very distinct functions [42, 92, 96]. Similarly, the motor system can be further separated into a cerebello-cortical and a basal-cortical loop with different patterns of involvement [97, 98]. The difficulty to define a network does not yield for primary networks only. For instance, it has been argued that the fronto-parietal network could be further partitioned into two subnetworks subserving attention and working memory, respectively, [99], while the working memory network itself could be further broken down in two, with one subnetwork mediating attentional selection and another one rather underlying language functions [100]. Networks are not exclusive from each other either. Mesulam [18] refers to transmodal nodes that connect various neurocognitive networks. For instance, activation of some fronto-parietal regions is observed during different cognitive tasks [101]; are these regions transmodal or part of a subnetwork that has a specific function? Similarly, the insular cortex is typically a multimodal association area that is not specifically activated by auditory stimuli. However, as reported in Section 4, recent papers have consistently classified it as belonging to a so-called auditory system. As evidenced by Figure 1, there are also some overlapping between networks, and voxels can be simultaneously classified as belonging to different networks. What is the function of such regions? Could this overlapping between networks be related to synchronization through distinct frequency-bands [35]—if such a phenomenon is indeed visible through fMRI BOLD imaging? Regarding the influence of a task on a network, an issue that has not received much attention yet, studies have shown that networks could indeed be influenced by the processing of a task, either during the task [86] or even after it [102]. It is hence not unrealistic to suspect that processing of a task might also modify the very structure of some networks.

Another cogent question is the relationship between networks as detected by fMRI data analyses and those mentioned in the literature. Networks extracted from fMRI are the consequence of the optimization of a mathematical criterion whose link to neuroscience is, at the very least, not obvious. While some results have been rather successfully related to the neurocognitive literature (e.g., attentional network), other results are more complex to interpret. Some networks extracted seem to share commonalities with some of the subtypes described by Mesulam [18] (e.g., the motor network; cf. Section 2), while others seem to be rather related to Mesulam [18]'s neurocognitive networks (e.g., the attentional network). Besides, the union of all reported networks (e.g., by sICA) does not include the whole brain. Some brain regions are then excluded from the functional networks organization of the brain. Why so? Globally, the criterion used for network extraction might make the methods sensitive to some functions or types of connections. For instance, top-down and bottom-up influences have distinct features [25, 34, 36–38]. Can they be detected equally well by existing methods?

Apart from these difficulties, there has also been evidence of variability across healthy subjects [80, 103] that could be explained by many factors, such as development and/or age [104, 105], and, in general, all forms of plasticity [106, 107]. Pathologies, for example, stroke [108, 109] or tumors [110–113], render the issue even more complex. Some studies have shown that certain pathologies can have network-specific effects: behavioral deficits in spatial neglects for the fronto-parietal network [114]; epilepsy [115] and Alzheimer's disease [116] for the default-mode network. Nonetheless, these results must be used with caution, for it is not clear yet whether they truly reflect a change in neuronal properties or, as, for example, in grade II glioma, a mere modification of the metabolic and vascular properties of the surrounding tissues.

### 5.2. BOLD fMRI imaging

Use of BOLD fMRI as a way to investigate large-scale networks relies on three successive assumptions, namely, that information exchanges between neurons is related to synchronies, synchronies to the BOLD contrast, and the BOLD contrast to the fMRI data effectively measured.

Synchronies are the blueprint of communication between regions [17, 39, 117–120] and, as such, should be strongly related to large-scale networks. A challenging issue is to determine the exact relationship between the spatial distribution and interaction pattern of regions within a large-scale network on the one hand and, on the other hand, the spatial and frequential distribution of oscillations.

Another issue is the connection between neuronal activity/synchrony and the appearance of a BOLD signal. While much still needs to be unraveled as to the connection between neuronal synchronies and the BOLD signal, it now seems more and more accepted that a sustained change in neuronal activity is likely to entail a relative change in the BOLD level [121–124], even though the exact relationship is expected to be rather complex [125].

 Still, the BOLD signal is only a fraction of the total signal that is acquired in fMRI, a signal that is not exempt from many kinds of artifacts [126–128]. In particular, some physiological processes (e.g., cardiac, respiratory, or movement-related) induce spurious effects that contaminate the BOLD signal in the whole brain [129, 130]. Such artifacts are predominant in certain regions of the brain, such as the basal arteries for cardiac activity or the interfaces between cerebrospinal fluid pools and brain tissus for breathing and head movements. This origin-dependent predilection implies a spatial structure of the noise. Some network detection algorithms may hence recognize voxels influenced by the same spatially structured artifact as meeting the requirement for strong temporal coherence and, hence, assign them to a common structure. This feature has been successfully used by ICA techniques to provide efficient noise separation and removal techniques [66, 131, 132]. Yet, the issue arises when structures induced by noise are wrongly interpreted as functional networks; their detection and removal is hence of very high importance. The fundamental question, while examining spatial structures with a similar temporal behavior, is “do we measure neurally induced signal or consequences of physiological processes [133]?” Even though our understanding of the potential artifacts that can contaminate the BOLD fMRI signal improves, the consequences of many potential sources of structured noise have barely been mentioned, let alone investigated. For instance, it is believed that some mechanisms related to the regulation of blood flow (e.g., through the level of CO_2_ in the blood) could induce coherent changes in BOLD signal throughout the brain—giving birth to an effect likely to be identified as a functional network. In fact, such an effect has been used to explain the presence of the default-mode network in fMRI [134, 135]. Now, whether these regions are wrongfully classified as belonging to a common functional network because their voxels are corrupted by the same artifact, or whether they are actually regions that drive the physiological response is a matter that remains to be solved.

### 5.3. Data analysis

Many questions remain open regarding what methodologies to apply to extract functional networks. We here quickly discuss issues related to the choice of a model, the redundancy of fMRI signals, the necessity to provide both individual and group analyses, the importance of result representation, and the validation of fMRI results with other modalities.

Procedures used to investigate networks are usually based on mathematical methods that have been discovered independently of the field of fMRI data analysis. Their behavior is hence not guided by cognitive but mathematical considerations. While it can be accepted that most methods are general enough to be applied to a wide variety of problems, they still require a careful assessment of how best to adapt them to the issue at hand. We believe that the major point to cope with here is “how do methods code for segregation and integration? Does it make sense?” A relevant approach could be to try to derive out consistence requirements from cognitive consideration of what an “ideal” method should be able to do: quantify integration between voxels, regions, or networks using the same principled measure (such as the multiple correlation coefficient [136] or integration [7, 137]); differentiate between direct and indirect information exchanges (such as partial correlation [19, 72, 138–141]); discriminate causality from simple co-occurrence (such as Granger causality [138, 142–144]). Some methods are able to deal with one aspect of the problem, but none has been proposed to answer all these questions simultaneously.

Besides, investigation of large-scale networks face a very interesting problem, namely that of determining the spatial precision under which data should be considered as segregated and over which they should be said to be integrated. While it is obvious that neighboring voxels share a great deal of information, methods that model and summarize the behavior of a whole network with one single time course clearly oversimplify the problem and discard a lot of cogent information. Bellec et al. [80] proposed a statistical model that provides a critical distance that separates segregation and integration. Voxel clustering is another attempt to deal with that issue. However, the parameters that characterize the clustering coarseness are set a priori, when they should be determined by the intrinsic properties of the data and allowed to vary across the brain (e.g., between subcortical and cortical structures, which have distinct characteristic spatial extents). This step allows one also to reduce the dimensionality of the data. At least for this reason, it is a crucial step, because network investigation requires multivariate analyses that are computationally very demanding (the computational burden roughly exponentialy increase with the number of regions).

In neurocomputing, models investigating issues very similar to that of large-scale networks have already been developed [145–148]. However, most methods developed so far for effective connectivity, such as structural equation modeling (SEM) [149–152], dynamical causal modeling (DCM) [153–155], or generative models—including neural mass models [145, 156] and large-scale neural models [147, 157–159]—, have been of little use to the investigation of extended large-scale networks, since their intrinsic complexity prevent them from modeling systems with that many degrees of freedom (but see, [160]).

Methods originating from graph and/or network theories might prove more adapted to such problems [161–163], since they provide global quantification of structures that, besides from being ubiquitous [164, 165], are not unlike some models of brain networks. Using such methods, brain networks have been shown to exhibit small-world [70, 166–170] and scale-free [171, 172] features. The fact that networks simultaneously exhibit both properties has strong structural [104, 173] and functional [174, 175] implications.

Being able to devise methods that can deal with both individual and group analyses is also an important issue. At the individual level, it is essential to assess the significance of the different networks [80, 85, 176]. With procedures of increasing complexity, nonparametric resampling procedures [177], mostly used in the context of the GLM so far [178], might be appealing [179]. At the group level, one seeks to determine invariant networks across subjects. This has been done by either considering a model for the group [76] or solving the problem at an individual level and then performing clustering [79]. Validation of such methods have to be developed; a first step in this direction has been proposed by Calhoun et al. [180].

Once results have been produced, representing the results becomes a key issue. Consider for instance functional connectivity as measured by marginal correlation. Though computationally tractable on a large-scale network of *N* units, even for *N* large, such a method generates *N*(*N*−1)/2 correlation coefficients (e.g., 4950 for as few as 100 voxels/regions; 19900 for 200; 499 500 for 1000). Simply representing these on a graph as is commonly done [72, 140, 181] would prove impossible to read, let alone to interpret. Procedures that summarize the information have to be proposed; these can rely on PCA/MDS [71, 79, 182]; they could also use other representational techniques [183, 184].

Last, but not least, an essential point to validate and better understand the large-scale network approach in fMRI is the comparison with results form other imaging modalities or areas of neuroscience, such as electrophysiology [34–38], electroencephalography (EEG) or magnetoencephalography (MEG) [39, 125, 185, 186], and diffusion tensor imaging (DTI) [187–190]. To be able to efficiently compare results from different imaging modalities, it is essential to better understand how each modality images the activity of large-scale brain networks. In this perspective, providing a unified generative and/or statistical model for several of these modalities would be of the utmost importance, granting access to multimodal in vivo imaging of the brain in action.

## Figures and Tables

**Table 1:** Literature summary of the different networks found in network investigation of fMRI data. Methods—HC: hierarchical clustering, SOM: self-organizing map algorithm, ICA: independent component analysis. Networks—M/SM: motor/sensorimotor, V: visual, A: auditory, DM: default mode, dAtt: dorsal attentional, vAtt: ventral attentional, EC: executive control. Networks found are denoted by “X.” (*) In addition to primary cortices (sensorimotor, visual, and auditory), the clusters shown by Cordes et al. [63] were essentially bilateral single regions (thalami, fusiform gyri, and frontal gyri) which were parts of different networks of reference. (†) Except for the sensorimotor system, the networks identified by Peltier et al. [61] were not properly labeled; the spatial organizations of the maps shown seemed similar to the attentional networks. (‡) The results presented by Calhoun et al. [73] were partial, mentioning the extraction of other networks that they did not show neither comment; another study on similar datasets showed that the sensorimotor and the dorsal attentional networks might be detected too [74].

Task	Reference	Method	Network						
			M/SM	V	A	DM	dAtt	vAtt	EC
At rest	[61]	HC	X	X	X		X(*)	X(*)	
	[63]	SOM	X				(†)	(†)	
	[73]	ICA	X	X	X	X			
	[75]	ICA	X	X	X	X	X		X
	[76]	ICA	X	X	X	X	X	X	X
	[77]	ICA	X	X		X	X	X

Blocked visual	[78]	ICA	X(‡)	X	X		X(‡)		
	[79]	ICA		X		X	X		

Blocked motor	[80]	LSNI	X			X	X		

**Table 2:** Example of extended large-scale networks extracted in fMRI at rest. Peak foci corresponding to the six networks identified using spatial ICA and a hierarchical clustering approach similar to that of [79] on a group of 20 healthy subjects acquired at rest—M/SM: motor/sensorimotor, V: visual, DM: default mode, dAtt: dorsal attentional, vAtt: ventral attentional, EC: executive control.